# Biocompatibility Assessment of Polydimethylsiloxane for Vitreous Substitution Application in Relation to Physicochemical Properties

**DOI:** 10.3390/polym18131597

**Published:** 2026-06-26

**Authors:** Diba Grace Auliya, Mutiara Septiani, Risdiana Risdiana

**Affiliations:** 1 Department of Physics, Faculty of Mathematics and Natural Sciences, Universitas Padjadjaran, Jl. Ir. Soekarno Km 21 Jatinangor, Sumedang 45363, West Java, Indonesia; 2 Department of Chemistry, Faculty of Mathematics and Natural Sciences, Universitas Padjadjaran, Jl. Ir. Soekarno Km 21 Jatinangor, Sumedang 45363, West Java, Indonesia; mutiara23030@mail.unpad.ac.id

**Keywords:** biocompatibility, cytotoxicity, emulsification, polydimethylsiloxane, stability, vitreous substitution

## Abstract

Polydimethylsiloxane (PDMS) has long been utilized as a vitreous humour substitute in the treatment of retinal detachment. To address increasing clinical demands, PDMS synthesis has been explored, yielding a range of viscosities of PDMS with favourable properties for this application. While various formulations and synthesis routes for developed PDMS have been reported in previous studies, an evaluation of their biocompatibility and relationship to physical properties has not yet been reported. However, the biocompatibility of this biomaterial is a critical determinant of its long-term performance. Accordingly, this study aims to evaluate the biocompatibility of PDMS and its relationship with physical properties through a comprehensive assessment that correlates polymer synthesis parameters, physicochemical properties, storage stability, emulsification resistance, and cytotoxicity. The samples tend to be stable during a five-month storage period. No signs of emulsification were observed when the samples were exposed to the emulsifiers. All samples exhibited no cytotoxic effect through the resazurin assay. Collectively, these findings suggest that synthesized PDMS possesses favourable biocompatibility and physicochemical stability, supporting its potential as a vitreous humour substitute.

## 1. Introduction

Abnormalities in the vitreous humour, the transparent gel-like structure filling the posterior chamber of the eye, are frequently associated with severe ocular disorders such as retinal detachment and visual impairment [1,2]. These conditions often require vitrectomy, a surgical procedure in which the natural vitreous body is removed and replaced with an artificial vitreous substitute to maintain retinal attachment and preserve visual function [3]. An ideal vitreous substitute should possess physicochemical and biological properties similar to those of the native vitreous humour, including optical transparency, appropriate refractive index, suitable viscosity for surgical manipulation, long-term stability, and excellent biocompatibility without including inflammatory or toxic responses. In addition, the material should resist emulsification, remain chemically stable during storage, and be economically accessible for widespread clinical use [1,3,4,5].

Among materials currently employed in vitreoretinal surgery, silicone oil (SO) remains the only clinically approved long-term vitreous substitute. SO is widely used due to its chemical inertness, optical transparency, and adjustable viscosity, which allows surgeons to select formulations suitable for different clinical conditions [6]. Commercial SOs are typically classified into low-, medium-, and high-viscosity categories, with higher viscosities generally associated with improved tamponade stability and longer intraocular residence time [1,7,8]. Despite these advantages, SO tamponade is not free from complications. One of the most critical challenges is emulsification, which leads to the formation of oil droplets that may migrate into ocular tissues and cause secondary complications such as inflammation, glaucoma, and keratopathy [9,10]. These complications have motivated continuous efforts to improve the physicochemical stability and biocompatibility of silicone-based vitreous substitutes [10,11].

Polydimethylsiloxane (PDMS), a linear silicone polymer composed of repeating –Si–O– backbone units with methyl side groups, constitutes the primary component of commercial SO. PDMS is typically synthesized via ring-opening polymerization (ROP) of cyclic siloxane monomers, such as octamethylcyclotetrasiloxane (D4), thereby enabling precise control of molecular weight and viscosity by adjusting reaction parameters. Previous studies have demonstrated that synthesis conditions, including catalyst concentration, chain terminator ratio, and reaction time, strongly influence the resulting polymer chain length and thus the physicochemical properties of PDMS [12,13,14]. However, most existing studies have focused primarily on optimizing synthesis conditions or characterizing physical properties, while comprehensive evaluations that link polymer synthesis, physicochemical characteristics, and biological performance remain relatively limited.

In addition to physicochemical stability, the biocompatibility of vitreous substitute materials is a critical determinant of their clinical safety and long-term performance. Biocompatibility refers to the ability of a material to interact with biological systems without eliciting adverse cellular or tissue responses. For silicone-based vitreous substitutes, emulsification resistance and cytotoxicity represent two essential indicators of biocompatibility [11]. Emulsification is influenced by interfacial properties such as surface tension and viscosity, which determine the stability of the oil–aqueous interface under mechanical or biochemical disturbances [9,15]. Meanwhile, cytotoxicity assays provide important information regarding potential cellular responses to the material [16]. Although several studies have independently investigated emulsification behavior or cytotoxic effects of PDMS, integrated assessments that simultaneously relate physicochemical properties, interfacial stability, and biological response are still scarce.

Another important aspect that remains underexplored is the development of locally synthesized PDMS in regions where commercial products are predominantly imported. The limited availability and high cost of imported PDMS may restrict clinical accessibility, particularly in developing countries [12,14]. Therefore, developing PDMS materials through scalable synthesis approaches while ensuring their physicochemical and biological suitability represents both a scientific and practical challenge.

In this study, we report the synthesis of PDMS with controlled viscosity ranges using an ROP approach and evaluate its potential as a vitreous substitute through an integrated biocompatibility assessment framework. The study systematically investigates the relationships between polymer synthesis parameters, physicochemical properties, storage stability, emulsification resistance, and in vitro cytotoxicity. By correlating these factors within a unified evaluation strategy, this work aims to provide a deeper understanding of the physicochemical property–biocompatibility relationship of PDMS materials for vitreous substitution applications. The results provide important insights into the design of stable and biocompatible silicone-based vitreous substitutes and highlight the potential of locally synthesized PDMS as a cost-effective alternative for ophthalmic applications.

## 2. Methods

**Figure 1:**
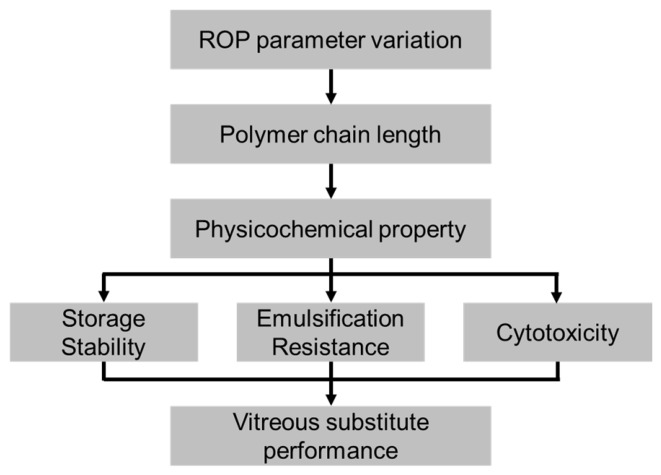
Sequential research stages within the integrated biocompatibility framework.

The methodology of this study was structured as a sequential series of research stages, as outlined in the integrated biocompatibility framework presented in Figure 1. The study began with the variation of ROP synthesis parameters to control polymer chain length, followed by evaluation of key physicochemical properties, including viscosity and surface tension. These physical properties were subsequently used to assess storage stability and were then correlated with emulsification resistance to evaluate material stability under relevant conditions. Subsequently, biocompatibility was evaluated through emulsification behaviour and cytotoxicity assays to determine suitability for biomedical applications. Finally, the overall performance as a vitreous substitute was assessed by integrating physicochemical and biological results.

**Figure 2:**
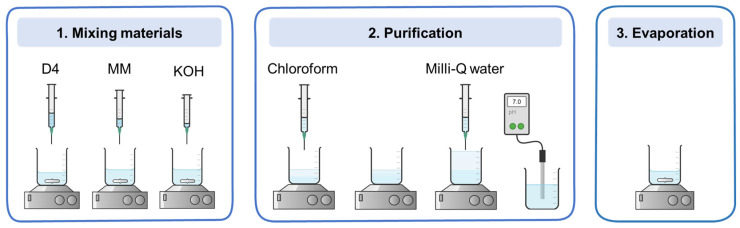
Synthesis procedure involving material mixing, purification, and evaporation.

Low-, medium-, and high-viscosity PDMS samples were synthesized from D_4_ through the ROP method following the previously reported procedure [17]. Hexamethyldisiloxane (MM) and potassium hydroxide (KOH) were used as the chain terminator and catalyst, respectively. The amount of monomer, chain terminator, and KOH, along with the synthesis temperature and time, were used as controlled parameters. The synthesis process began with mixing the materials and continues with purification and evaporation, resulting in PDMS. These processes were explained in detail in Figure 2.

The mixed materials include: 31.2 mL of D_4_ monomer (98%, Alfa Chemical, Zhengzhou, Henan, China), 12 mL of MM (99.5%, Sigma Aldrich, Darmstadt, Germany), and 0.140 mL of 0.6 M KOH (Merck, Darmstadt, Germany). A 100 mL beaker was preheated to 150 °C, after which D_4_, MM, and KOH were sequentially introduced in the specified order. The reaction mixture was stirred at 300 rpm and maintained at 150 °C for 35, 40, or 42 min to obtain the targeted PDMS viscosity, sequentially for low-, medium-, and high-viscosity PDMS. Samples were purified to a neutral pH after washing with chloroform and Milli-Q water. Chloroform and Milli-Q water were added at sample-to-solvent volume ratios of 1:1 and 1:2, respectively. PDMS samples were obtained after chloroform had completely evaporated. Then, the samples were coded as S1, S2, and S3 based on their viscosity category as shown in Table 1.

Physicochemical characterizations were carried out to measure the viscosity, refractive index, surface tension, and functional groups of the PDMS samples. Then, samples were stored at 2–8 °C for five months to determine the stability of the physical properties of the material during storage time. The physical properties were re-evaluated in this study, including viscosity, refractive index, and surface tension. Meanwhile, ^1^H-NMR spectroscopy (400 MHz) was employed to identify the characteristic functional groups of the synthesized PDMS.

Emulsification resistance of the samples was evaluated by mixing them with infusion fluid and albumin protein as the emulsifier. The infusion fluid and albumin protein are components found naturally in the eye. Albumin protein, the most abundant protein in the eye, was chosen due to its reported ability to act as an emulsifier agent [9,18]. The sample and emulsifier, in a 1:1 ratio, were placed in a cuvette and sonicated following the previous method [8,19]. Observations were made at 0, 3, and 30 min of sonication. The emulsified sample will form between the non-emulsified sample (top) and the emulsifier (bottom).

The toxicity of the samples was assessed through the resazurin cytotoxic method on CV-1 cells. CV-1 cells were employed as a standard mammalian cell line for preliminary cytotoxic screening due to their stable proliferation and widespread use in biomaterial safety evaluation. This test was chosen as an initial screening method in assessing the toxicity of materials due to its advantages of speed, sensitivity, simplicity, affordability, and good correlation with other cytotoxic test results [16,20,21,22,23]. The testing procedure comprised cell culture, sample dilution, incubation, and reagent addition. The cell culture was incubated until approximately 70% confluence was achieved. The samples were diluted into eight concentration variants using the media solvent. A total of 100 μL of each sample and controls were placed in a 96-well plate containing cells, followed by incubation for 48 h. Each well received 100 μL of PrestoBlue^®^ reagent and was incubated until a colour change became evident.

**Figure 3:**
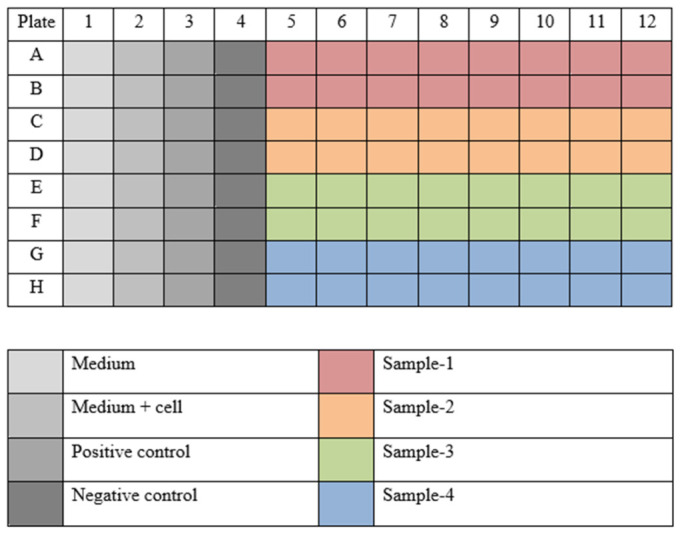
Well plate layout for the resazurin cytotoxicity test.

The colour changes in the reagent occurred when the blue compound resazurin was reduced to the highly fluorescent red compound resorufin, with the absorbance value reflecting the cell viability. All incubation steps were conducted at 37 °C under 5% CO_2_ conditions. The positive control used in this test was 39, 48 μM of Cisplatin, while the negative control was 2% of DMSO solvent. The plate layout is presented in Figure 3, while the detailed concentrations of each sample are listed in Table 2.

## 3. Results and Discussion

### 3.1. Polymer Synthesis and Physicochemical Properties of PDMS

**Figure 4:**
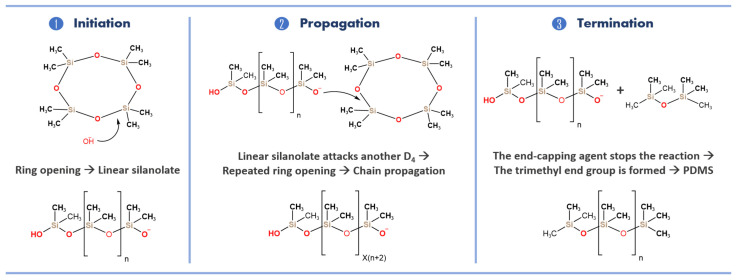
The ROP reaction scheme of D_4_, including initiation, propagation, and termination steps leading to PDMS formation.

PDMS was successfully synthesized through ROP of the cyclic siloxane monomer D_4_. The polymerization process proceeds through three fundamental stages: initiation, propagation, and termination, as shown in Figure 4. During the initiation stage, hydroxide ions originating from the KOH catalyst open the cyclic siloxane ring of D_4_, generating reactive silanolate intermediates. These intermediates subsequently propagate through successive Si–O bond formation, resulting in the growth of linear polymer chains [24,25]. Chain termination occurs when MM reacts with the active chain ends, effectively controlling the molecular weight of the resulting polymer [26]. Consequently, PDMS with certain properties was formed. The synthesis parameters were the factors influencing these differences in properties. The effects of individual synthesis parameters on the PDMS properties have been systematically described in previous studies [14,27,28,29].

**Figure 5:**
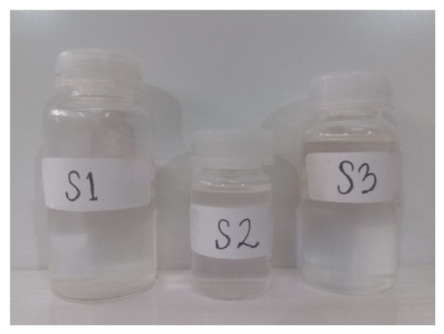
Transparent appearance of the synthesized PDMS samples.

**Figure 6:**
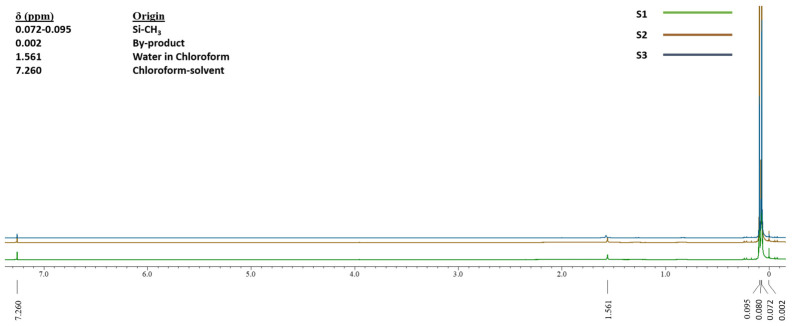
H-NMR spectra of samples S1, S2, and S3.

Key physical properties that play a crucial role in vitreous humour substitute application include viscosity, refractive index, and surface tension. Viscosity is related to the ease of manipulation during the injection process [30]. Refractive index affects diopters [7]. While surface tension stabilizes the shape and prevents emulsification [9]. The physical appearance of all synthesized PDMS samples is shown in Figure 5, and their physical properties are presented in Table 3. For comparative purposes, the physical properties of the synthesized PDMS and commercial SO are summarized in Table 4. The chemical identity of the synthesized PDMS was verified by NMR spectroscopy, as shown in Figure 6.

The viscosity of the synthesized PDMS samples increased with increasing reaction time, indicating progressive chain growth during polymerization. The longer the chain formed, the higher the viscosity produced [26]. As shown in Table 3, the obtained viscosities correspond to low (S1), medium (S2), and high (S3) viscosity categories. This trend is consistent with polymerization kinetics, where longer reaction times enable the formation of longer polymer chains and therefore higher resistance to shear deformation.

The measured refractive indices of the synthesized PDMS samples fall within the range typically reported for silicone-based vitreous substitutes. Optical compatibility is an essential requirement for vitreous replacement materials because variations in refractive index can affect the optical power of the eye [7]. The refractive index values obtained in this study correspond to diopter values in the range of +3.128 to +3.2211, close to those of commercial SO (+3.377 to +3.397), suggesting that the synthesized PDMS can maintain acceptable optical performance for intraocular applications.

Surface tension represents another critical parameter influencing the stability of SO within the ocular environment. The synthesized PDMS samples exhibited surface tension values between 20 and 21.5 mN/m, slightly higher than those reported for commercial SO (approximately 19–20 mN/m). Higher surface tension increases the energetic barrier required to create new oil–water interfaces, thereby reducing the likelihood of droplet formation under mechanical or biochemical disturbances. Consequently, the relatively elevated surface tension observed in the synthesized PDMS may contribute to enhanced resistance to emulsification, a major complication associated with SO tamponade [6]. Therefore, the physicochemical properties of PDMS were recognized as the primary factor influencing its stability [31].

The ^1^H-NMR spectra in Figure 6 exhibited a strong peak at 0.072–0.095 ppm, corresponding to the methyl protons attached to silicon (Si–CH_3_), which is the characteristic resonance of PDMS. The presence of this dominant peak confirms the formation of PDMS. Additional peaks observed at 1.56 and 7.260 ppm were attributed to residual water in the deuterated chloroform and the deuterated chloroform solvent, respectively [13]. A minor signal was also detected at 0.002 ppm, indicating the presence of a trace by-product. Collectively, the NMR data confirmed the chemical identity of all synthesized samples as PDMS.

### 3.2. Storage Stability of Synthesized PDMS

Long-term stability is an essential requirement for vitreous substitute materials because physicochemical changes during storage may influence their clinical performance. To evaluate storage stability, the synthesized PDMS samples were stored at 2–8 °C for five months, and their properties were periodically re-evaluated as shown in Table 5. These properties include viscosity, refractive index, and surface tension, as their crucial role in vitreous humour substitution application.

The viscosity values of the samples showed only minor variations over the storage period. Samples S1 and S2 exhibited slight increases in viscosity, whereas sample S3 showed a marginal decrease. These small variations may be attributed to minor structural rearrangements within the polymer chains or measurement fluctuations rather than significant chemical degradation. Importantly, the viscosity categories of all samples remained unchanged, indicating that the polymer network maintained its structural integrity during storage.

Similarly, the refractive index values exhibited negligible changes throughout the storage period. The refractive index plays a key role in determining the speed at which light travels through the material relative to a vacuum. Furthermore, since refractive index is closely related to molecular structure and density, the stability of this parameter suggests that no substantial changes in the bulk chemical structure or density of the PDMS occurred during storage [32].

Surface tension remained constant for all samples throughout the five-month storage period. This observation indicates that the interfacial properties of the synthesized PDMS are highly stable, which is particularly important for preventing emulsification during storage and clinical use. The combined stability of viscosity, refractive index, and surface tension suggests that the synthesized PDMS possesses robust physicochemical stability suitable for long-term storage prior to clinical application. A longer observation period is needed to determine the optimal storage and usage times for PDMS.

### 3.3. Emulsification Resistance

Emulsification is one of the most critical challenges associated with PDMS tamponade in vitreoretinal surgery. The formation of emulsified droplets may lead to the migration of PDMS into ocular tissues, potentially causing secondary complications such as inflammation, glaucoma, or corneal damage [9,10]. Therefore, evaluating the resistance of PDMS to emulsification is essential for assessing its suitability as a vitreous substitute.

**Figure 7:**
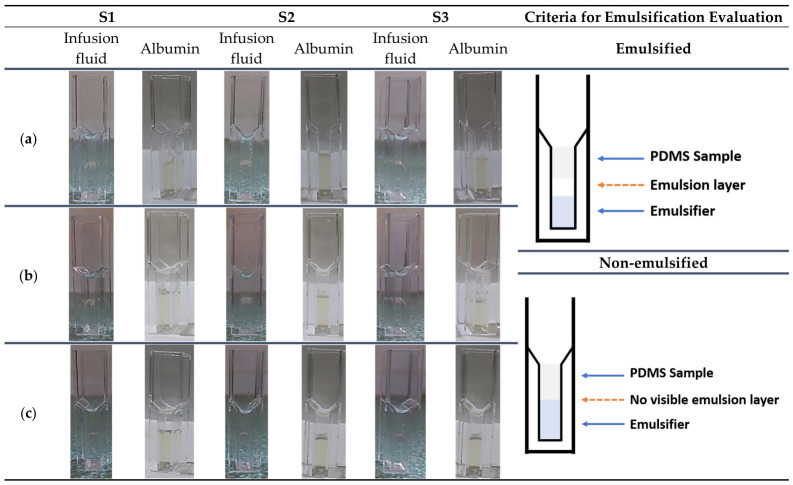
Emulsification test at (**a**) 0 min, (**b**) 3 min, and (**c**) 30 min of sonication.

Previous studies evaluated emulsification resistance using serum and plasma, components found in blood, as emulsifiers [8,19]. In this study, the emulsifiers were specifically selected to represent components abundantly present in the intraocular environment, with infusion fluid and albumin protein serving as representative [4,6]. Infusion fluid is an ionized solution designed to resemble the ionic composition of biological fluids. Albumin, which constitutes a significant fraction of ocular proteins, can act as a biological surfactant capable of lowering the interfacial tension between oil and aqueous phases [4,6,30]. Mechanical agitation induced by sonication further promotes droplet formation, providing a stringent condition for evaluating emulsification stability. Emulsification test results before and after sonication with emulsifiers are shown in Figure 7.

The experimental results demonstrated that none of the PDMS samples exhibited visible emulsification after sonication for up to 30 min. These results align with the finding that there were no significant changes in the samples after the storage period. The absence of a distinct emulsified layer suggests that the synthesized PDMS maintains strong interfacial stability in the presence of biological emulsifiers. The physical stability of the material is a key parameter in determining its resistance to emulsification [10,15].

From a physicochemical perspective, emulsification behavior is governed by a combination of interfacial tension and viscosity. Higher surface tension increases the thermodynamic barrier for droplet formation, while higher viscosity suppresses droplet breakup during mechanical agitation [6]. Therefore, the relatively high surface tension measured in the synthesized PDMS samples plays an important role in stabilizing the oil phase and preventing droplet dispersion. In addition, the increasing viscosity from S1 to S3 further enhances resistance to shear-induced droplet formation. The synergistic effect of these parameters likely explains the strong emulsification resistance observed in the present study.

### 3.4. Cytotoxicity Evaluation

Cytotoxicity assays provide an important preliminary evaluation of the biological safety of materials by assessing their potential effects on cell viability and proliferation. In this study, the cytotoxicity of the synthesized PDMS samples was evaluated using the resazurin-based assay on CV-1 cells. The resazurin assay, also known as alamar blue, is widely used for cytotoxicity screening due to its sensitivity, simplicity, and ability to quantitatively measure metabolic activity in living cells [16]. In this method, viable cells reduce the non-fluorescent resazurin dye into the fluorescent compound resorufin, resulting in a colour change from blue to pink. The intensity of this colour change is directly proportional to cellular metabolic activity and therefore reflects cell viability [20].

**Figure 8:**
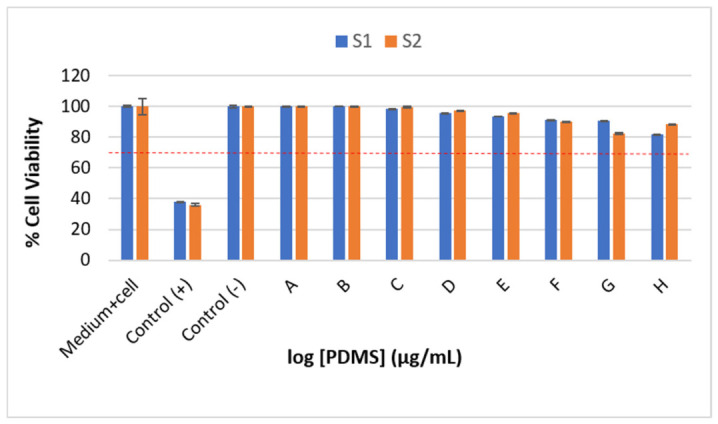
Cell viability results of samples S1 and S2 at different concentrations. Error bars represent the standard error of the mean (SEM). The dashed red line indicates the 70% viability threshold.

**Figure 9:**
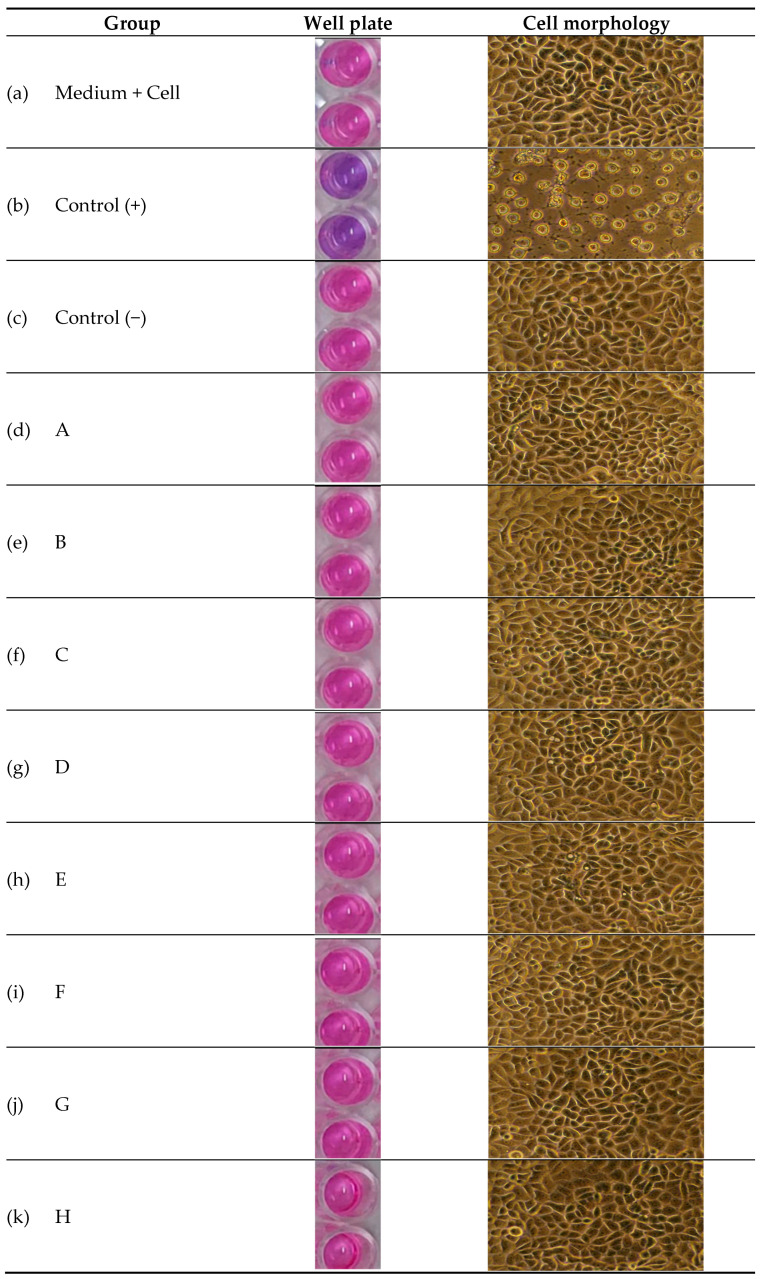
Well plate appearance and CV-1 cell morphology for sample S1. Panels (**a**–**k**) represent medium + cell, positive control, negative control, and experimental group (A–H), respectively. All micrographs were acquired at 20× magnification.

**Figure 10:**
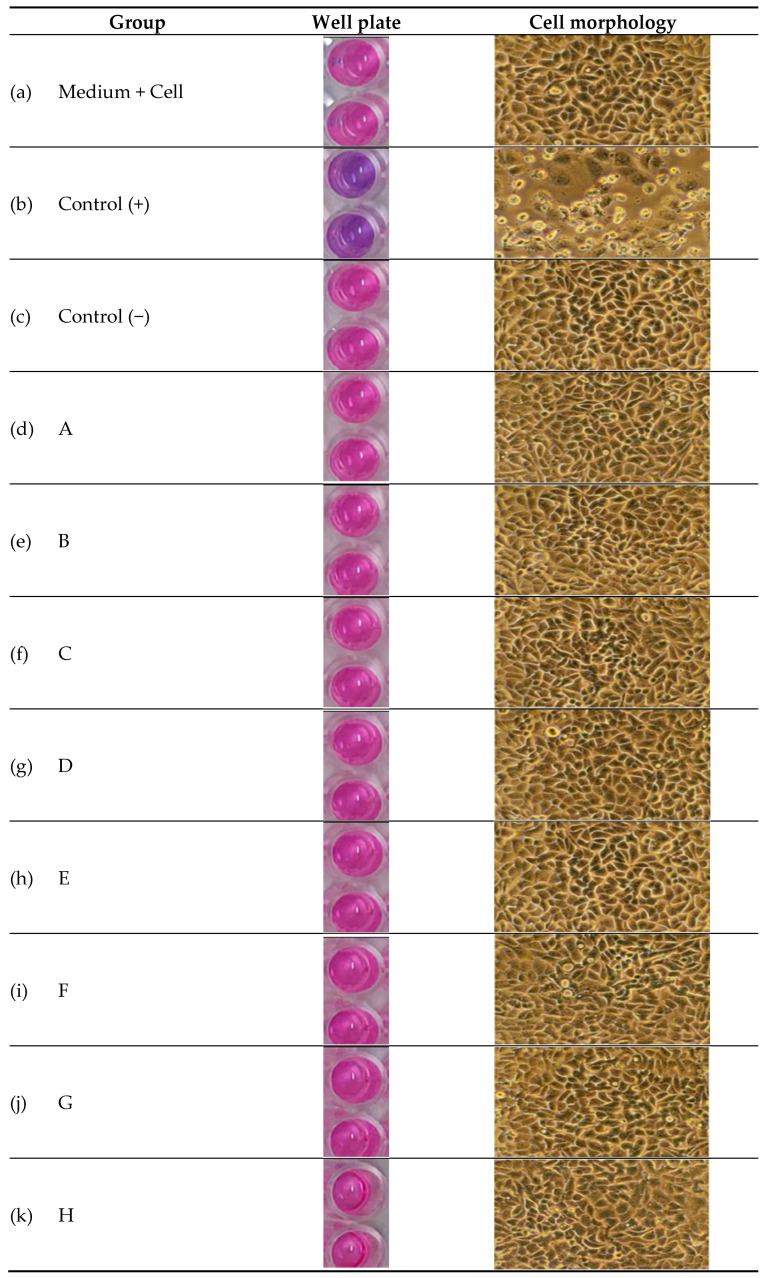
Well plate appearance and CV-1 cell morphology for sample S2. Panels (**a**–**k**) represent medium + cell, positive control, negative control, and experimental group (A–H), respectively. All micrographs were acquired at 20× magnification.

The resazurin cytotoxicity test was conducted on S1 and S2 samples with various concentrations as a general approach to evaluate the effect of PDMS samples on the viability of living cells. The viability of living cells is described by absorbance measurement. Living cells will give pink and fluorescent staining. These results were compared with the control material to evaluate the potential toxicity of PDMS to living cells. The resazurin test results showed that the higher the PDMS concentration, the lower the absorbance. Consequently, cell viability decreased. However, the test results showed that cell viability was still above 80% for both samples, as shown in Figure 8. The well plate and CV-1 cell morphology for each sample concentration are shown in Figure 9 and Figure 10.

The results indicate that both tested PDMS samples maintained cell viability above 80% across all tested concentrations (81.60 ± 0.08% to 100 ± 0.29%), with low SEM values indicating a small margin of error. According to commonly accepted cytotoxicity criteria, materials that maintain cell viability above 70% are generally considered non-cytotoxic [33]. Therefore, a dashed red line representing the 70% viability threshold has been included in Figure 8 to facilitate the interpretation of cytotoxicity results. In addition to quantitative viability measurements, morphological observations further confirmed the absence of cytotoxic effects such as swelling, cell rupture, and cell death. Cells exposed to PDMS samples retained normal morphology comparable to the negative control, whereas the positive control exhibited clear signs of cellular damage. The antiproliferation test showed a large value of >500,000 μg/mL for both samples, indicating low effectiveness of the samples in inhibiting cell growth. These findings suggest that the synthesized PDMS does not induce adverse cellular responses under the tested conditions. The high biocompatibility observed in this study is consistent with the chemical inertness and hydrophobic nature of PDMS, which limit its interaction with cellular membranes and intracellular components.

### 3.5. Property–Biocompatibility Relationship

The combined results obtained in this study highlight the importance of the property–biocompatibility relationship in determining the suitability of PDMS as a vitreous substitute material. The synthesis parameters used in the ROP process influence the molecular weight and chain structure of PDMS, which in turn determine key physicochemical properties such as viscosity, refractive index, and surface tension.

These physicochemical properties directly affect the material’s interfacial behaviour and biological performance. Higher viscosity enhances mechanical stability and suppresses droplet fragmentation during agitation, while elevated surface tension increases resistance to interfacial instability and emulsification. Together, these parameters contribute to improved resistance to emulsification, one of the major causes of complications in silicone oil tamponade.

Furthermore, the absence of cytotoxic effects demonstrates that the optimized physicochemical properties of the synthesized PDMS do not compromise biological compatibility. Therefore, the integration of physicochemical characterization, emulsification testing, and cytotoxicity evaluation provides a comprehensive assessment framework for evaluating vitreous substitute materials.

Overall, the results demonstrate that controlling PDMS synthesis parameters can produce materials with an optimal balance between physicochemical stability, emulsification resistance, and biological safety. This integrated evaluation approach provides valuable insights for the rational design of silicone-based vitreous substitutes with improved clinical performance. The results suggest that locally synthesized PDMS can serve as a promising and potentially cost-effective alternative to commercial silicone oils for ophthalmic applications. However, further advances in biocompatibility assessment, including a longer-term stability evaluation, biomolecular interaction, and in vivo validation, are also recommended to ensure the complete biocompatibility information for this material.

## 4. Conclusions

This study demonstrates the successful synthesis of PDMS with controlled viscosity ranges through a ROP approach and evaluates its potential application as a vitreous substitute through an integrated physicochemical and biological assessment. The synthesized PDMS samples exhibited physicochemical properties comparable to those of commercial SO, including suitable viscosity and refractive index, while presenting slightly higher surface tension that may contribute to enhanced interfacial stability.

The storage stability evaluation revealed that the viscosity, refractive index, and surface tension of the PDMS samples remained largely unchanged over a five-month storage period, indicating strong physicochemical stability. Furthermore, the emulsification tests showed that all samples maintained excellent resistance to emulsification in the presence of biological emulsifiers, suggesting that the combination of relatively high surface tension and appropriate viscosity provides favourable interfacial stability under mechanically induced conditions.

The in vitro cytotoxicity assessment demonstrated high cell viability across all tested concentrations, confirming that the synthesized PDMS does not induce adverse cellular responses and possesses good biological compatibility. These findings collectively highlight the importance of controlling polymer synthesis parameters in tailoring the physicochemical properties that govern the interfacial stability and biological safety of silicone-based vitreous substitutes.

Overall, this study establishes a property–biocompatibility relationship for synthesized PDMS materials and provides an integrated evaluation framework for assessing their suitability as vitreous substitutes. The results suggest that locally synthesized PDMS can serve as a promising and potentially cost-effective alternative to commercial silicone oils for ophthalmic applications. Future studies involving extended stability evaluation, biomolecular interaction analysis, and in vivo validation will be essential to further confirm the long-term clinical performance of this material.

## Figures and Tables

**Table 1:** Sample identification and coding based on viscosity value.

Sample Code	Viscosity Category
S1	Low viscosity
S2	Medium viscosity
S3	High viscosity

**Table 2:** Sample concentration for the resazurin cytotoxicity test.

Code	Sample Concentration (μg/mL)
A	3906.25
B	7812.50
C	15,625
D	31,250
E	62,500
F	125,000
G	250,000
H	500,000

**Table 3:** Physical properties of PDMS samples.

Sample	η (Pa·s)	*n*	ɣ (mN/m)
S1	1.00	1.3989	21
S2	2.12	1.4001	20
S3	3.60	1.3997	21.5

**Table 4:** Physical property comparison between synthesized PDMS and commercial SO.

Property	Synthesized PDMS	Commercial SO [14,26]
Viscosity (Pa·s)	1.00–3.60	0.9–5.5
Refractive Index	1.3839–1.4001	1.404
Surface Tension (mN/m)	20–21.5	19–20
Emulsification	None observed	Reported in the clinical case

**Table 5:** Properties of PDMS samples after storage for 0, 1, and 5 months.

Sample	η (Pa·s)			*n*			ɣ (mN/m)		
	0	1	5	0	1	5	0	1	5
S1	1.00	1.00	1.13	1.3989	1.3989	1.4001	21	21	21
S2	2.12	2.12	2.18	1.4001	1.4001	1.4008	20	20	20
S3	3.60	3.60	3.47	1.3997	1.3997	1.3989	21.5	21.5	21.5

## Data Availability

The original contributions presented in this study are included in the article. Further inquiries can be directed to the corresponding authors.

## Abbreviations

The following abbreviations are used in this manuscript:

D_4_	Octamethylcyclotetrasiloxane
MM	Hexamethyldisiloxane
PDMS	Polydimethylsiloxane
ROP	Ring opening polymerization
SEM	Standard Error of the Mean
SO	Silicone oil
